# An observational cohort study of interstitial lung abnormalities (ILAs) in a large Japanese health screening population (Kumamoto ILA study in Japan: KILA-J)

**DOI:** 10.1186/s12890-023-02455-y

**Published:** 2023-06-08

**Authors:** Kazuya Ichikado, Hidenori Ichiyasu, Kazuhiro Iyonaga, Kodai Kawamura, Noritaka Higashi, Takeshi Johkoh, Kiminori Fujimoto, Jun Morinaga, Minoru Yoshida, Katsuhiko Mitsuzaki, Moritaka Suga, Naoya Tanabe, Tomohiro Handa, Toyohiro Hirai, Takuro Sakagami

**Affiliations:** 1 Division of Respiratory Medicine, Saiseikai Kumamoto Hospital, 5-3-1 Chikami, Chuo-ku, Kumamoto, 861-4101 Japan; 2 Department of Respiratory Medicine, Kumamoto University, 1-1-1 Honjo, Chuo-ku, Kumamoto, 860-8556 Japan; 3 Department of Respiratory Medicine, Kumamoto Red Cross Hospital, 2-1-1 Nagamineminami, Higashi-ku, Kumamoto, 861-8039 Japan; 4 Japanese Red Cross Kumamoto Health Management Care Center, 2-1-1 Nagamineminami, Higashi-ku, Kumamoto, 861-8528 Japan; 5 Department of Radiology, Kansai Rosai Hospital, 3-1-69 Inabaso, Amagasaki, Hyogo 660- 8511 Japan; 6 Department of Radiology, Center for Diagnostic Imaging, Kurume University School of Medicine, Kurume University Hospital, 67 Asahi-machi, Kurume, Fukuoka 830-0011 Japan; 7 Department of Clinical Investigation (Biostatistics), Kumamoto University Hospital, 1-1-1 Honjo, Chuo-ku, Kumamoto, 860-8556 Japan; 8 Saiseikai Kumamoto Hospital, Center for Preventive Medicine, 5-3-1 Chikami, Chuo-ku, Kumamoto, 861-4101 Japan; 9 Department of Respiratory Medicine, Graduate School of Medicine, Kyoto University, Kawahara 54, Shogoin, Sakyo-ku, Kyoto, 606-8507 Japan

**Keywords:** Interstitial lung abnormality (ILA), Idiopathic pulmonary fibrosis, Progressive pulmonary fibrosis (PPF)

## Abstract

**Background:**

Interstitial lung abnormalities (ILAs) are subtle or mild parenchymal abnormalities observed in more than 5% of the lungs on computed tomography (CT) scans in patients in whom interstitial lung disease was not previously clinically suspected and is considered. ILA is considered to be partly undeveloped stages of idiopathic pulmonary fibrosis (IPF) or progressive pulmonary fibrosis (PPF). This study aims to clarify the frequency of subsequent IPF or PPF diagnosis, the natural course from the preclinical status of the diseases, and the course after commencing treatment.

**Methods:**

This is an ongoing, prospective, multicentre observational cohort study of patients with ILA referred from general health screening facilities with more than 70,000 annual attendances. Up to 500 participants will be enrolled annually over 3 years, with 5-year assessments every six months. Treatment intervention including anti-fibrotic agents will be introduced in disease progression cases. The primary outcome is the frequency of subsequent IPF or PPF diagnoses. Additionally, secondary and further endpoints are associated with the efficacy of early therapeutic interventions in cases involving disease progression, including quantitative assessment by artificial intelligence.

**Discussion:**

This is the first prospective, multicentre, observational study to clarify (i) the aetiological data of patients with ILA from the largest general health check-up population, (ii) the natural course of IPF or PPF from the asymptomatic stage, and (iii) the effects and outcomes of early therapeutic intervention including anti-fibrotic agents for progressive cases of ILA. The results of this study could significantly impact the clinical practice and treatment strategy for progressive fibrosing interstitial lung diseases.

**Trial registration number:**

UMIN000045149.

**Supplementary Information:**

The online version contains supplementary material available at 10.1186/s12890-023-02455-y.

## Background

An interstitial lung abnormality (ILA) is defined as “incidental identification of non-dependent abnormalities on computed tomography, including ground-glass or reticular abnormalities, lung distortion, traction bronchiectasis/bronchiolectasis, honeycombing, and non-emphysematous cysts involving at least 5% of a lung zone in individuals in whom interstitial lung disease is not suspected [1–3]. The clinical importance of ILAs has been increasingly recognised as early and asymptomatic lesions in cases of progressive fibrosing interstitial lung diseases (ILDs) [4, 5]. Furthermore, the presence of ILAs is a prognostic factor in ILD, lung cancer, and chronic obstructive pulmonary disease (COPD). This is because ILAs are subcategorised as non-fibrotic and fibrotic, with fibrotic abnormalities thought to represent early or mild pulmonary fibrosis, resulting in a higher risk of complications in lung cancer or COPD [1–3, 6]. Although an asymptomatic period in which ILAs can be detected during high-resolution computed tomography (HRCT) examinations is likely to precede a pulmonary fibrosis diagnosis, the natural history of progression from ILA stage to overt disease remains unknown [4, 5]. This challenge has remained unresolved with progressive pulmonary fibrosis (PPF) as a newly defined pathological concept characterised by progressive fibrosis as well as IPF [5, 7]. Furthermore, the effect of early anti-fibrotic drug therapies for ILA on progressed disease stages is unknown. However, anti-fibrotic drugs have effectively prevented the decline of forced vital capacity (FVC) with a potential for improved IPF or PPF prognoses [8, 9].

The frequency of ILA and predictors of ILA progression have been identified in large studies on COPD cases and populations at risk of cardiovascular diseases [3, 6]. In addition to age, smoking history, body mass index (BMI), and genotype being predictive background factors for ILA progression [3, 6, 10], HRCT findings and patterns of ILA have also been identified as progression and prognostic factors [10–12]. However, there is limited data on ILA in many general check-up populations [12]. Hence, this study aims to clarify the natural course from ILA or the asymptomatic stage in patients subsequently diagnosed with IPF or PPF and the course and outcome after the introduction of therapeutic interventions. Additionally, this study also aims to clarify the frequency and progression index of ILA by analysing ILA cases screened from general health screening facilities with the largest number of examinees.

## Methods/design

### Study design

This is a prospective, multicentre observational cohort study on ILAs diagnosed by respiratory specialists from general health screening facilities in Kumamoto Prefecture, Japan. Other details of the study’s design are as follows:

- Presence or absence of invasion: Mild invasion (no new needle insertion with plasma blood sampling at enrolment:)
- Presence or absence of intervention: No intervention.
- Presence or absence of use of samples collected from the human body: Yes (plasma and serum are stored).
- Positioning of the study: observational study.
- Randomisation: None.

### Participant characteristics

#### Study population

Eligible candidates are not only cases with suspected ILA on chest CT at health check-up facilities, but also cases confirmed by the health check-up facilities to have reticular shadows at the costophrenic angles on chest X-ray with or without the presence of fine crackles on auscultation, assuming no history of interstitial pneumonia. Training sessions will be held for physicians in the screening check-up facilities participating in the study to confirm the study participants using any of these three findings. Cases with either of these findings in the screening check-up facilities are suspected cases of ILA. The study will enrol approximately 500 patients from > 70,000 examinees in general health check-up facilities, annually screened by chest radiography, with fine crackles on auscultation and/or CT findings. Participants will be enrolled over 3 years and followed up for 5 years. The primary outcome is the evaluation of the aetiological data of preclinical patients who are subsequently diagnosed with IPF or PPF. This study has been designed according to the Strengthening the Reporting of Observational Studies in Epidemiology Statement guidelines. Patients or the public were involved in the design, conduct, reporting, or dissemination plans of our research.

#### Inclusion criteria

Patients with one or more of the following findings during check-ups will be suspected of having ILAs which are slightly modified in the Fleischner Society criteria [1]: (i) fine reticular shadows at the costophrenic angles on chest radiography, (ii) fine crackles on auscultation, and (iii) lower predominant subpleural reticular shadows on CT findings. Patients suspected to have ILAs at a screening facility will be referred for further examination in any of the three teaching hospitals (Kumamoto University Hospital, Japanese Red Cross Kumamoto Hospital, and Saiseikai Kumamoto Hospital) where pulmonologists work. Patients diagnosed with ILAs confirmed using HRCT who provided informed consent will be enrolled into the study’s database.

This study defines ILAs as subtle or mild parenchymal abnormalities observed in > 5% of the lungs during CT examination of patients in whom ILD has not been previously clinically suspected.

The final diagnosis for ILA based on the diagnosis using HRCT by the pulmonologists in each case will be confirmed in institutional conferences of the three teaching hospitals. This can be strengthened by having a concordant diagnosis by a radiologist who is an expert in respiratory diseases or by concordance with more than two pulmonologists who reviewed the records of each patient who met the inclusion criteria.

#### Exclusion criteria

The following are exclusion criteria: (i) patients who refuse to participate in the study after reading the explanation, (ii) patients with acute or subacute progressive interstitial pneumonia, (iii) patients who have difficulties undergoing the periodic follow-up due to their distant places of residence, (iv) cases that are assessed as inappropriate for the study by medical attendants, (v) cases incorporated into intervention studies, such as clinical trials that are not allowed to participate in observational studies.

### Data collection and management

#### Primary endpoint

The primary endpoint is the frequency of subsequent IPF or PPF diagnoses.

#### Secondary endpoints

The secondary endpoints are (i) frequency of ILA cases among all general health examiners and (ii) frequency of fibrotic hypersensitivity pneumonitis and collagen vascular diseases related to interstitial pneumonia in patients diagnosed with PPF.

#### Further endpoints

The progression rate of ILA cases will be examined using HRCT. In addition, IPF cases involving patients with ILA will be evaluated for (i) the rates of decline in FVC, percentage diffusing capacity for carbon monoxide (%DLco), and 6-min walk distance; and (ii) the progression rate of the ILD-Gender–Age–Physiology (ILD-GAP) GAP Index. Rate of decline in 6-min walk distance in cases of IPF diagnosed from ILA patients.

The progression rate of HRCT findings in IPF cases involving patients with ILA will be assessed by comparing the visual and quantitative assessments using artificial intelligence-based quantitative CT (AIQCT) [13]. In addition, the progression rate of HRCT findings in PPF cases involving patients with ILA will be assessed by comparing the visual and quantitative assessments using AIQCT. Furthermore, the proportion of HRCT findings showing progression in IPF or PPF cases before and after therapeutic interventions will be assessed by comparing the visual and quantitative assessments using AIQCT.

Other endpoints to be assessed include: (i) the association between HRCT progression indices and functional indices or clinical outcomes in patients with IPF or PPF, (ii) the rate of FVC decline before and after anti-fibrotic drug therapy in IPF and PPF cases involving patients with ILA, (iii) mortality rates of IPF from the asymptomatic stage, (iv) incidence of acute exacerbation in IPF and PPF cases from the asymptomatic stage, (v) the frequency of respiratory complications (pneumonia, lung cancer, and others) requiring inpatient treatment of patients with IPF or PPF from the asymptomatic stage, (vi) rate of decline in %DLco before and after therapeutic intervention in PPF cases involving patients with ILA, and (vii) the output score of image AI to assess suspicions of interstitial pneumonia from chest X-rays [14].

#### Data collection

There is insufficient evidence to support the cost-benefit ratio of screening asymptomatic individuals. Nonetheless, this study’s testing plan follows the 2020 recommendations of the Fleischner Society [1]. Details of data that will be collected during the scheme are described in the Online Supplementary Materials (Supplementary Table 1).

Patients will be enrolled after confirming that they fulfil all inclusion criteria without meeting any exclusion criteria and they provide informed signed consent. In addition, the following data will be collected and registered at enrolment: (i) sex, date of birth, height, weight, and BMI; (ii) reason for a referral from screening facilities; (iii) smoking history, environmental history (presence or absence of dust exposure, contact with birds, and occupational history), and family history of interstitial pneumonia; (iv) comorbidities, including diabetes mellitus, coronary artery disease, gastroesophageal reflux, cardiovascular diseases other than coronary artery disease, and lung cancer; and (v) presence of fine crackles on auscultation, chest radiography, and HRCT findings and patterns based on international guidelines [7].

The following physical examination results will be collected every six months: oxygen saturation (SpO_2_) levels and dyspnoea scale score (modified Medical Research Council), pulmonary function tests, blood gas analysis, and a 6-min walk test. Other tests with results include biological assessments (including blood count and renal and hepatic functions), autoantibody screening blood sampling, serum interstitial pneumonia markers (Krebs von den Lungen-6 (KL-6), surfactant protein-D (SP-D)), ILD-GAP index, and echocardiography results. Suspected diagnosis and diagnostic accuracy of IPF based on ontology will be recorded after discussing the multidisciplinary diagnosis (MDD) via a web conference involving the three institutions and each institution’s diagnosis.

Patients that show any signs of disease progression, including the appearance of clinical symptoms, decline in lung function, or exacerbation of HRCT findings evaluated by visual assessment and AIQCT [13], will be considered for a full work-up and the introduction of drug therapy. The detailed examination will include bronchoalveolar lavage (BAL), transbronchial lung cryobiopsy, or surgical lung biopsy. After registration, subsequent pulmonary complications requiring hospitalisation, such as acute exacerbation, pneumonia, pneumothorax, or lung cancer, will be recorded.

Furthermore, it will be recorded if long-term oxygen therapy is needed in cases of progression despite appropriate medication during the follow-up years. When therapeutic intervention is introduced, the assessment plan is reset, and regular assessments are carried out during the period from the introduction of treatment (Supplementary Table 2).

#### Management of enrolled participants

Data and images obtained during routine observation will be shared with the three medical centres via electronic data capture (EDC) and the imaging cloud. Based on the data, each of the three medical centres performs a case-specific institutional MDD and registers it in the EDC. HRCT images will also be evaluated independently by two experienced chest radiologists (T.J. and K.F.) for the first 100 cases at the start of enrolment to check the concordance rate of HRCT findings. Starting from the 101st case after the concordance rate is confirmed, each one will be assessed by one radiologist. Cases in which cryobiopsy or surgical lung biopsy is performed are referred to a pulmonary pathologist for pathological diagnosis, and the results are recorded on the EDC. In addition, cases registered at the three screening medical centres are regularly web-case-conferenced between facilities. Furthermore, the facility MDD and the web-conference MDD will be recorded on the EDC.

#### Timing of the start of treatment

Regular mandatory check-ups are carried out every six months by a registered researcher at a medical screening centre and progression is assessed according to the Progressive Fibrosing-Interstitial Lung Disease progression criteria [15]. If deemed to be a progression, the facility MDD will also be consulted and a decision will be made to introduce therapeutic agents.

### Statistical analysis

#### Sample size calculation

There are ≥ 70,000 examinees yearly for the Japanese Red Cross Kumamoto Hospital Health Care Center and the Center for Preventive Medicine of Saiseikai Kumamoto Hospital. The number of participants suspected of having ILAs was approximately 300 at each centre in 2018. Therefore, the total number of participants with ILAs is assumed to be approximately 600 per year.

According to the previous data, the number of cases will be underestimated by 10% because approximately 10% of the medical check-ups that require detailed tests for ILAs did not undergo the subsequent detailed examinations. Therefore, the maximum number of suspected ILAs is expected to be 500 cases annually.

Continuous variables will be expressed as means and standard deviation (SD), or medians and interquartile ranges (IQRs), and categorical variables for each group will be expressed as the number of subjects and percentages. The inter-observer variation in the presence or absence of HRCT findings will be analysed using the weighted kappa statistic and classified as follows: poor (κ = 0–0.20), fair (κ = 0.21–0.40), moderate (κ = 0.41–0.60), substantial (κ = 0.61–0.80), and almost perfect (κ = 0.81–1.00). Inter-observer variation regarding the extent of the HRCT findings was assessed using Spearman’s rank correlation coefficient. The HRCT scores assigned by the two independent observers are compared using the Bland–Altman method. If the HRCT patterns or scores do not agree between the two radiologists; one of the patterns or scores is adopted by consensus.

All analyses with ILA progression as the outcome variable are performed using logistic regression, where ILA progression was dichotomised with progression defined as probable or definite progression, and regression defined as no change, probable regression, and definite regression using subjective visual estimation as well as an objective AIQCT. Multivariable analyses will be adjusted for variables such as age, sex, and smoking status. In addition, a Cox proportional-hazards model has been created to assess treatment efficacy, considering the immortal period as a time-dependent covariate.

Furthermore, morbidity and mortality related to respiratory disease will be compared between groups using chi-squared or Fisher’s exact tests. Finally, variation in the global extent of ILA on HRCT examination between each evaluation timepoint will be compared between groups using the Mann–Whitney U or Wilcoxon tests. The extent of ILA according to a visual semi-quantified score or a quantified score by AI will be assessed by the Wilcoxon test for trends over time per case. The degree of progression for each diagnosed group of IPF or PPF will be compared with these scores using the Mann–Whitney U test.

Clinical laboratory evaluations will be expressed as mean, median, and interquartile range for each evaluation timepoint, and boxplots will be created for graphical representation. In addition, percentages of abnormal clinical laboratory results will be compared between the groups using chi-squared or Fisher’s exact tests. No interim data analysis has been planned.

## Discussion

This is the first prospective, multicentre, observational study to clarify (i) the aetiological data of patients with ILA from a large general health check-up population, (ii) the natural course of IPF or PPF from the asymptomatic stage, (iii) the effects and outcomes of early therapeutic intervention including anti-fibrotic agents for progressive cases of ILA, (iv) the new prognostic markers of ILA progression which are critical to better determine patients at a higher risk of IPF and PPF, and (v) the effectiveness of newer therapies for IPF on earlier ILA identification where therapeutic attenuation of functional decline will be more impactful than at the latter end stages of established IPF.

### Aetiological data of patients with ILA from the general health check-up population

The AGES-Reykjavik study, an epidemiological study including patients aged ≥ 40 years at risk of cardiovascular lesions, reported that 378 (7%) patients with ILAs on initial HRCT were observed from 5320 participants. Furthermore, 327 of 378 patients who underwent subsequent CT scans were evaluated for ILA progression [6, 10]. The AGES-Reykjavik study reported that 238 (73%) of 327 participants eventually showed ILA progression over 5 years (median: 5.1 years). ILA progression was also assessed in 1867 participants in the Framingham Heart Study using serial CT scans [6, 11]. The study reported that 155 (8%) of 1867 participants showed ILAs on initial CT scans. Furthermore, 119 (76%) of the 155 participants showed ILA progression on serial CT scans during the 6-year follow-up period. In a CT lung cancer screening program, 41 (2.4%) of 1699 subjects had ILA on baseline CT and 10 (24.4%) of 41 patients with ILA were subsequently diagnosed ILD after more than 4 years [16].

Although these studies did not describe the specific diagnoses in cases that showed ILA progression, the mortality rate from pulmonary fibrosis in the group with ILA was reported to be as high as 47% in the AGES-Reykjavik study.

The prevalence of ILA in an Asian health screening cohort of 2765 patients was reported to be approximately 3% [13]. The present clinical study is supposed to include a large number of participants with ILAs observed on HRCT, especially in the largest population (> 210,000 examinees) of general health check-ups. Hence, the study’s primary and further endpoints will be achieved.

### Specific MDD for cases progressing from the ILA stage and response to early therapeutic intervention

It has been reported that 20% of ILA cases progress within 2 years, and > 40% progress within 5 years [1, 2]. As well as increasing age, smoking history, BMI, and increasing copies of *MUC5B* promoter polymorphism being predictive background factors for ILA progression [3, 6, 11], the fibrotic signs of lung distortion, traction bronchiolectasis/bronchiectasis, and honeycombing on HRCT imaging are considered to be crucial for the progression of ILA and poor prognosis [10, 12, 17, 18]. However, there has been little data about how many patients are subsequently diagnosed with IPF or PPF. Furthermore, the natural history of the asymptomatic phases corresponding to the ILA stages in these progressive fibrosing phenotypes has not been elucidated. A recent meta-analysis showed that anti-fibrotic treatment reduces the risk of all-cause mortality in progressive fibrosing phenotypes, including IPF [8, 9]. However, it is critical to determine whether the early identification and diagnosis of these fibrotic ILDs could lead to early treatment, resulting in a better prognosis [5]. The present study’s flow is to assess the ILA stage every 6 months. Once clinical progression is confirmed, a full examination will be considered, including BAL and biopsy, according to the 2022 International Guidelines, and the degree of confidence in the MDD. Clarification on the progression from ILA to an advanced disease stage and treatment responsiveness in the respective pathologies of IPF and PPF is expected.

### Indicators of functional impairment during the ILA phase

Functionally, ILA progression was associated with a greater FVC decline after adjustment for covariates when compared with participants without ILA [11]. Reductions in total lung capacity and reduced exercise capacities, such as a greater reduction in SpO_2_ values during the 6-min walk test or 6-min walk distance, have been also reported in the ILA group compared with those in the non-ILA group [19]. However, there are no data on how often functional impairments should be assessed during the ILA phase. Although differences in ILA prognosis have been reported according to different initial HRCT patterns [10, 12, 17, 18], differences in the progression of functional disabilities are also expected. Indicators of functional impairment during the ILA phase have not been fully investigated according to the initial HRCT patterns of the ILA. The present study hopes to clarify whether these functional test abnormalities in the ILA phase are observed at 6-month test intervals and whether there are differences in HRCT imaging patterns.

### Relationship between AI analysis for quantification of ILA on HRCT scans and clinical outcomes

AI and machine learning approaches have become increasingly important for quantifying and classifying ILA and fibrotic ILDs [13]. They will also help address the increasing demand for identifying the progression of ILA. In this study, quantitative assessments of ILA using AI will be combined with visual assessments that semi-quantify the visual extent of ILA in 5% increments, as well as the pattern of HRCT findings in line with the 2022 IPF international guidelines [7]. The presence or absence of progression will be assessed quantitatively using AI (AIQCT) and semi-quantitatively by visual assessment. The relationship between the degree of progression and HRCT pattern at enrolment and changes in lung function indices and serum markers will also be investigated.

### Study limitations

First, since almost all patients suspected to have ILAs are asymptomatic, they may not necessarily visit a teaching hospital after referrals. For example, previous data from one of the check-up facilities (The Center for Preventive Medicine of Saiseikai Kumamoto Hospital) shows that the rate of missing re-evaluation was approximately 10%. Second, patients may fail to attend the regular 6-month visits after consenting to participate in the study because they are asymptomatic. Finally, a viral infection pandemic, such as COVID-19 or flu, might also affect regular visits every 6 months. For example, during the COVID-19 pandemic, patients feared the infection and tended to withhold regular hospital visits.

## Electronic supplementary material

Below is the link to the electronic supplementary material.

Supplementary Material 1

Supplementary Material 2

## Data Availability

Data on the EDC database after the start of the study will be analysed annually and data to be published will be considered for data disclosure upon request for data use.

## Abbreviations

AI: artificial intelligence
AIQCT: artificial intelligence-based quantitative computed tomography
BAL: bronchoalveolar lavage
EDC: electronic data capture
HRCT: high-resolution computed tomography
ILA: interstitial lung abnormality
ILD-GAP: score, interstitial lung disease-gender-age-physiology score
IPF: idiopathic pulmonary fibrosis
KL-6: Krebs von den Lungen-6
MDD: multidisciplinary discussion diagnosis
PPF: progressive pulmonary fibrosis
SP-D: surfactant protein D
