# Supplementary material for: An observational cohort study of interstitial lung abnormalities (ILAs) in a large Japanese health screening population (Kumamoto ILA study in Japan: KILA-J)
Source: BMC Pulm Med. 2023 Jun 8;23:199. doi: 10.1186/s12890-023-02455-y (PMC10249548; doi:10.1186/s12890-023-02455-y)
Supplement: Supplementary file 2 — Supplementary Material 2 [file 12890_2023_2455_MOESM2_ESM.pdf]

Supplementary Table 2. Schedule of Activities after introduction of treatment

Time / Activities	Entry<br>Period of time allowed	6 Mo.<br>3- 9 Mo.	12 Mo.<br>9-15 Mo.	18 Mo.<br>15-21 Mo.	24 Mo.<br>21-27 Mo.	30 Mo.<br>27-33 Mo.	36 Mo.<br>33-39 Mo.	42 Mo.<br>39-45 Mo.	48 Mo.<br>45-51 Mo.	54 Mo.<br>51-57 Mo.	60 Mo.<br>57-63 Mo.	At the time of Discont inuation
Consent	○	—	—	—	—	—	—	—	—	—	—	—
Patient background (age, date of birth, gender, height, weight)	○	—	—	—	—	—	—	—	—	—	—	—
Reasons for referral from screening facilities	○	—	—	—	—	—	—	—	—	—	—	—
Family history of interstitial pneumonia	○	—	—	—	—	—	—	—	—	—	—	—
Smoking History (Brinkman Index)	○	—	—	—	—	—	—	—	—	—	—	—
Dust Exposure History	○	—	—	—	—	—	—	—	—	—	—	—
Occupational Art (organic solvents, organic dusts, inorganic dusts)	○	—	—	—	—	—	—	—	—	—	—	—
Comorbidities/pre-existing medical conditions (diabetes, coronary artery disease, cardiovascular diseases other than coronary artery disease, gastro-esophageal reflux)	○	—	—	—	—	—	—	—	—	—	—	—
Environmental history of bird-related exposure<br>(e.g. history of duvet use, poultry manure, stuffed birds, aviaries)	○	—	—	—	—	—	—	—	—	—	—	—
Housing history (e.g. age, sun exposure, mould)	○	—	—	—	—	—	—	—	—	—	—	—
Chest X-P	○	○	○	○	○	○	○	○	○	○	○	△
High-resolution CT	○	○	○	○	○	○	○	○	○	○	○	△
HRCT pattern<br>UIP<br>Probable UIP<br>Indeterminate for UIP<br>Alternative diagnosis	○	○	○	○	○	○	○	○	○	○	○	△

ILA pattern												
HP guideline<br>HRCT pattern<br>Non-fibrotic<br>Fibrotic<br>Typical<br>Compatible with HP<br>Indeterminate for HP	○	○	○	○	○	○	○	○	○	○	○	△
Extent of HRCT findings (visual assessment: 5% increments)	○	○	○	○	○	○	○	○	○	○	○	△
Extent of HRCT findings (assessed by AIQCT)	○	○	○	○	○	○	○	○	○	○	○	△
Blood gas analysis	○	—	○	—	○	—	○	—	○	—	○	△
6MWT	○	△	○	△	○	△	○	△	○	△	○	△
PFT FVC	○	○	○	○	○	○	○	○	○	○	○	△
%FVC	○	○	○	○	○	○	○	○	○	○	○	△
VC	○	○	○	○	○	○	○	○	○	○	○	△
%VC	○	○	○	○	○	○	○	○	○	○	○	△
%DLco	○	○	○	○	○	○	○	○	○	○	○	△
%DLco/VA	○	○	○	○	○	○	○	○	○	○	○	△
FEV1.0	○	○	○	○	○	○	○	○	○	○	○	△
FEV1.0%	○	○	○	○	○	○	○	○	○	○	○	△
Modified MRC	○	○	○	○	○	○	○	○	○	○	○	△
Cough, presence of fine crackles on auscultation	○	○	○	○	○	○	○	○	○	○	○	△
Autoantibody screening blood sampling	○	—	○	—	○	—	○	—	○	—	○	△
Specific autoantibody blood sampling	△	—	△	—	△	—	△	—	△	—	△	△
Presence of symptoms of connective tissue diseases	○	○	○	○	○	○	○	○	○	○	○	△
Diagnosis of interstitial lung disease	○	○	○	○	○	○	○	○	○	○	○	△
ILD-GAP score・stage	○	○	○	○	○	○	○	○	○	○	○	△
Diagnostic accuracy of IPF (≦90%Confident, 89-70% Provisional high confident, 69-50% Provisional low confident, 50%≧ Unclassifiable)	○	○	○	○	○	○	○	○	○	○	○	△

ECG	○	—	—	—	—	—	—	—	—	—	○	△
# blood biochemistry test	○	○	○	○	○	○	○	○	○	○	○	△
Serum marker(KL-6, SP-D, ACE（optional）, sIL-2R (optional)	○	○	○	○	○	○	○	○	○	○	○	△
Stored plasma and serum collection	○	—	—	—	—	—	—	—	—	—	○	△
Bronchoalveolar lavage(BAL)（optional）＊	△	△	△	△	△	△	△	△	△	△	△	△
Transbronchial cryobiopsy（optional）＊	△	△	△	△	△	△	△	△	△	△	△	△
Surgical lung biopsy （optional）＊	△	△	△	△	△	△	△	△	△	△	△	△
Presence or absence of acute exacerbations	—	○	○	○	○	○	○	○	○	○	○	○
New onset of pneumonia (infection)	—	○	○	○	○	○	○	○	○	○	○	○
Presence or absence of lung cancer complications	○	○	○	○	○	○	○	○	○	○	○	○
Presence of complications requiring inpatient treatment	—	○	○	○	○	○	○	○	○	○	○	○
Availability of home oxygen therapy	—	○	○	○	○	○	○	○	○	○	○	○
Reasons for discontinuation of the study/outcome	—	—	—	—	—	—	—	—	—	—	—	○

△: Optional at the discretion of the attending physician

#: Blood and biochemical tests: white blood cell count (monocyte fraction, monocyte count) red blood cell count, haemoglobin, haematocrit, platelet count, albumin, creatinine, AST, ALT, total bilirubin, ALP, gamma GTP, LDH, CRP

*:Conditionally recommended in individual cases according to international guidelines and should be considered when patient consent is obtained for testing for a definitive diagnosis.
